# Supplementary material for: Optical functionalization of human Class A orphan G-protein-coupled receptors
Source: Nat Commun. 2018 May 16;9:1950. doi: 10.1038/s41467-018-04342-1 (PMC5956105; doi:10.1038/s41467-018-04342-1)
Supplement: Supplementary file 1 — Supplementary Information [file 41467_2018_4342_MOESM1_ESM.pdf]

SUPPLEMENTARY INFORMATION

Optical functionalization of human Class A orphan G-protein coupled receptors

Morri et al.

This PDF file includes:

Supplementary Figures 1 to 8

Supplementary Tables 1 to 9

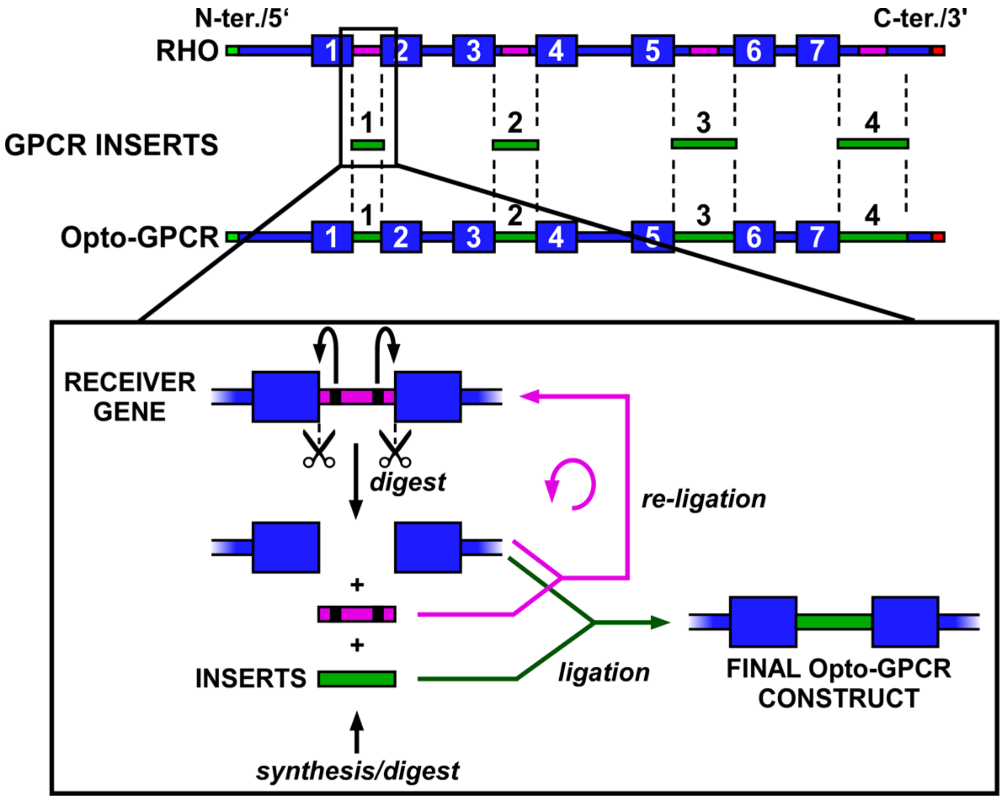

Supplementary Figure 1. High-throughput cloning strategy. The strategy is a modified form of Golden Gate cloning[1]. A rhodopsin (RHO) receiver gene harbors a non-coding genetic element (pink) at each intracellular element insertion site. These non-coding elements contain two recognition sites (black) for a TypeIIS restriction enzyme. Enzymes were BsaI (site 1: ICL1), BsmBI (site 2: ICL2), BpiI/BbsI (site 3: ICL3), and LguI/SapI (site 4: C-terminus). The recognition sites are oriented in opposite direction (black arrows) to achieve excision of the non-coding element during digest (scissors). After excision and when combined with coding inserts that were obtained by synthesis/digest, the receiver vector can either undergo religation with the non-coding genetic element (pink arrows, yields original receiver vector that will be redigested) or ligation with the inserts (green arrows, yields final construct that is not digested because no recognition sites are present). Temperature cycling allows obtaining high ratios of final construct to receiver gene (>9:1) and the reaction is in one tube with simultaneous ligation. Not shown are sticky end overhangs, VSV-G epitope at N-terminus (light green), and 1D4-epitope of rhodopsin at the C-terminus before the stop codon (red). Refer to **Supplementary Table 4** for detailed sequence design and **Supplementary Table 6** for reaction performance.

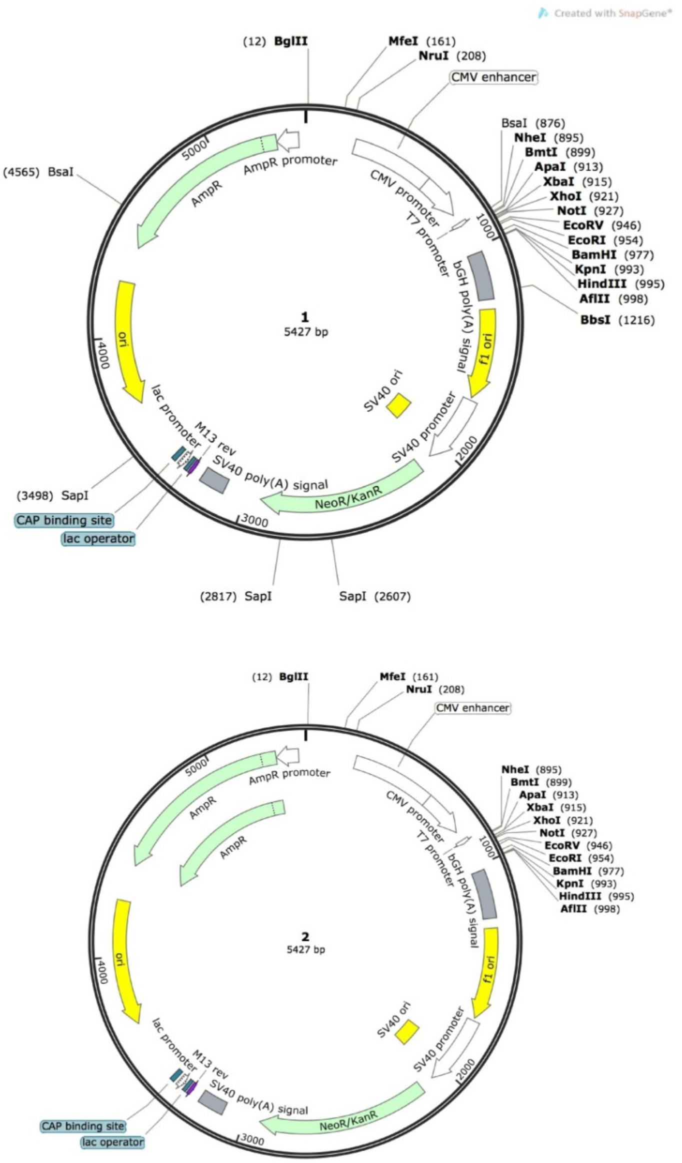

Supplementary Figure 2. Modified mammalian expression vector. (**1**) Map of the original pcDNA3.1(-) vector with six recognition sites of the TypeIIS restriction enzymes BsaI, BpiI/BbsI, and LguI/SapI. (**2**) Modified vector termed pcDNA3.1(-)-ΔREIIS after removal of all six sites using site-directed mutagenesis. Refer to **Supplementary Table 5** for vector sequence. The modified vector is available through Addgene.org.

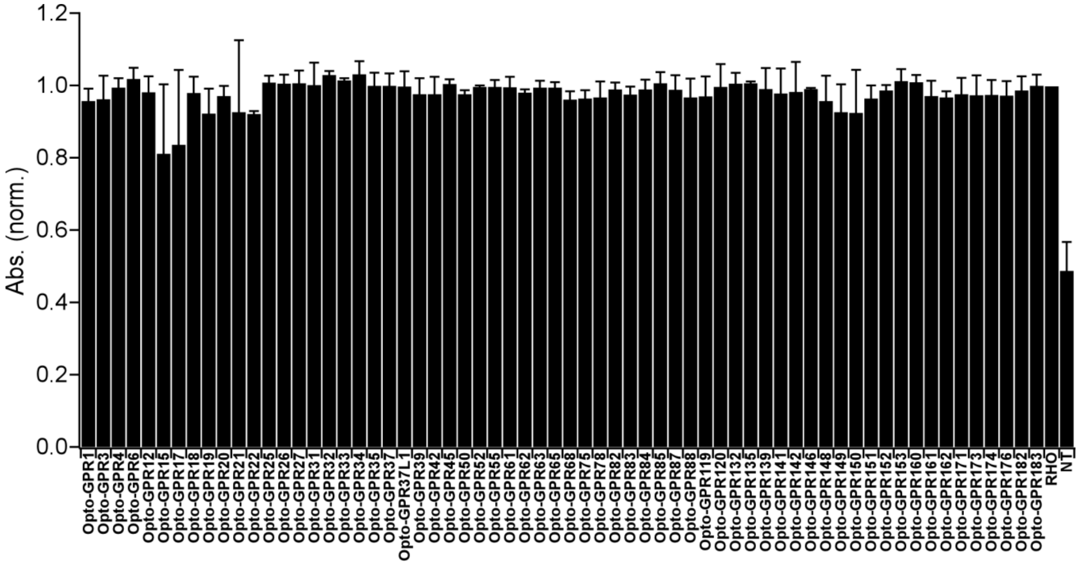

Supplementary Figure 3. Expression of chimeric receptors. Absorbance values from HEK293 cells transfected with chimeric receptors detected using an antibody directed against the extracellular N-terminal VSV-G epitope. Mean absorbance values (normalized to epitope-tagged rhodopsin (RHO)) $\pm$ s.d. (3 independent experiments) are shown. NT: Not transfected.

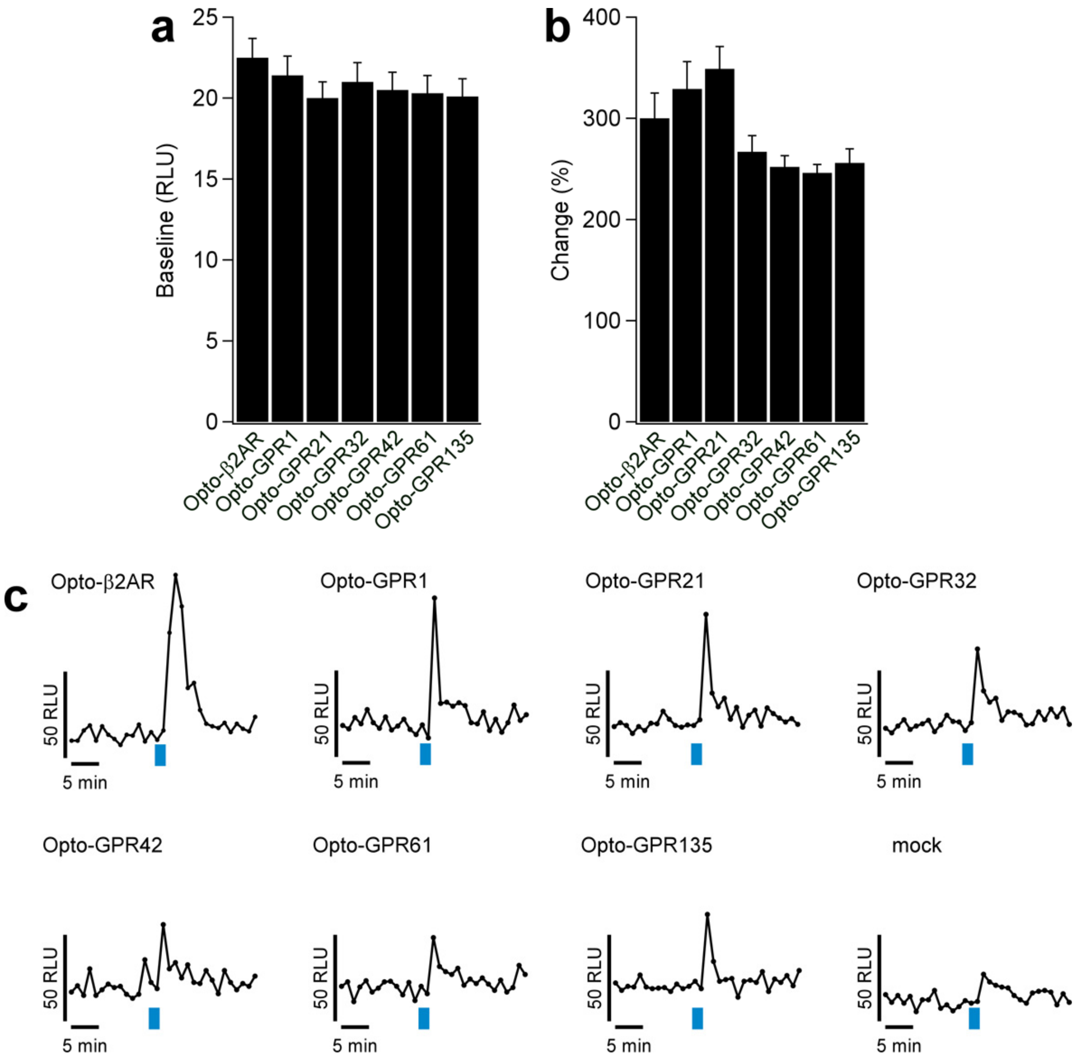

Supplementary Figure 4. Light-induced cAMP mobilization. (**a** and **b**) Baseline RLU (a) and relative responses upon light stimulation (b) of HEK293 cells transfected with chimeric receptors and a real-time sensor for intracellular cAMP. Mean RLU values ± s.e.m. (n=13-15 wells, 5 independent experiments) are shown. (**c**) Representative raw data traces (average traces of triplicate wells from the same experiment; blue bars indicate light stimulation).

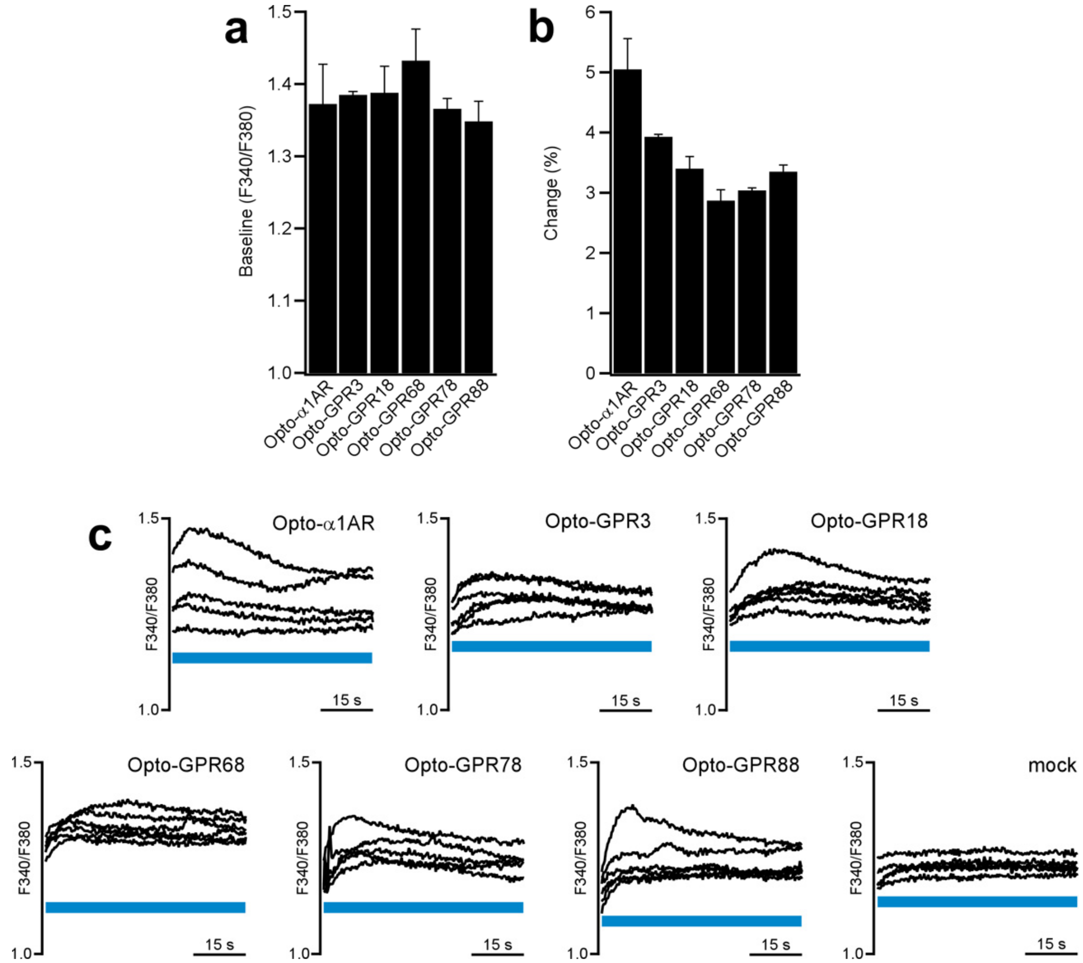

Supplementary Figure 5. Light-induced Ca^{2+} mobilization. (**a** and **b**) Baseline F340/F380 ratios (a) and relative responses upon light stimulation (b) of HEK293 cells transfected with chimeric receptors and labelled with a Ca^{2+}-sensitive dye (Fura2). Mean values ± s.d. (n=45-75 cells, 3-5 independent experiments) before and after stimulation are shown. (**c**) Representative raw data traces (5 individual cells; light was applied throughout the traces).

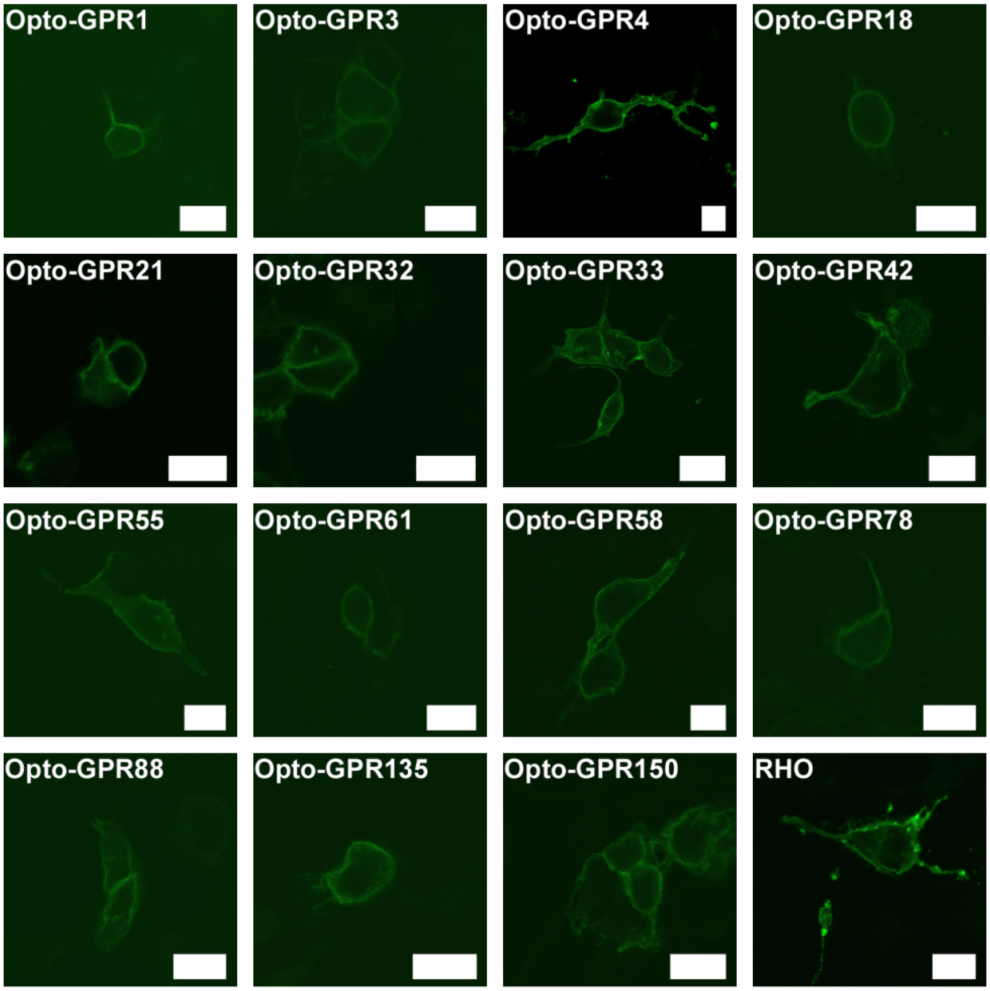

Supplementary Figure 6. Surface localization of chimeric receptors. Confocal microscopy images of HEK293 cells expressing chimeric receptors with induction or reduction of CRE, SRE-.L and SRE reporters detected using an antibody directed against the extracellular N-terminal VSV-G epitope. Scale bars are 10 μm.

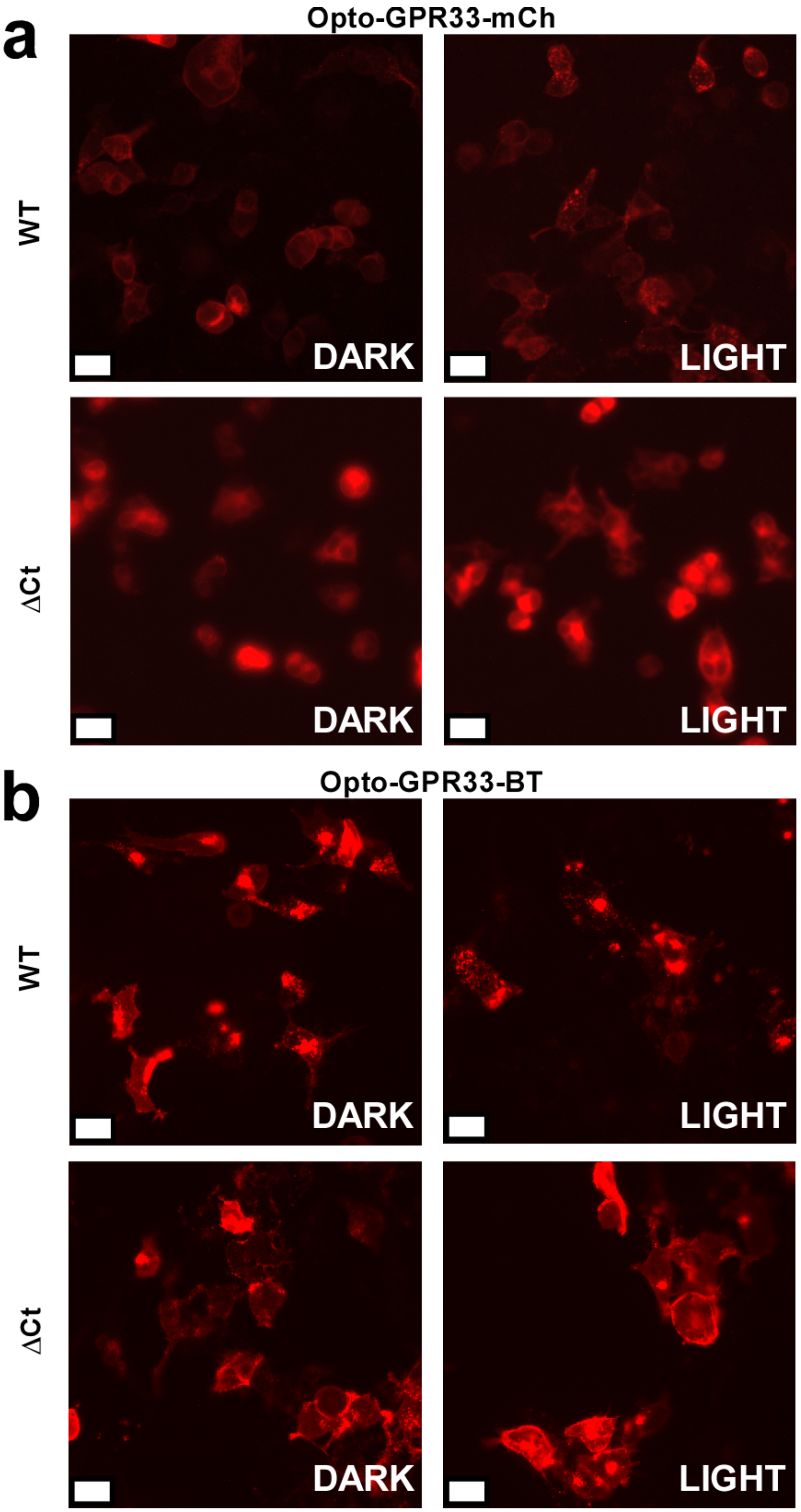

Supplementary Figure 7. Internalization of Opto-GPR33(ΔCt) detected as mCherry (mCh) fusion proteins (a) or by labelling of biotinylated receptors with fluorescent streptavidin (b). Scale bars are 10 μm.

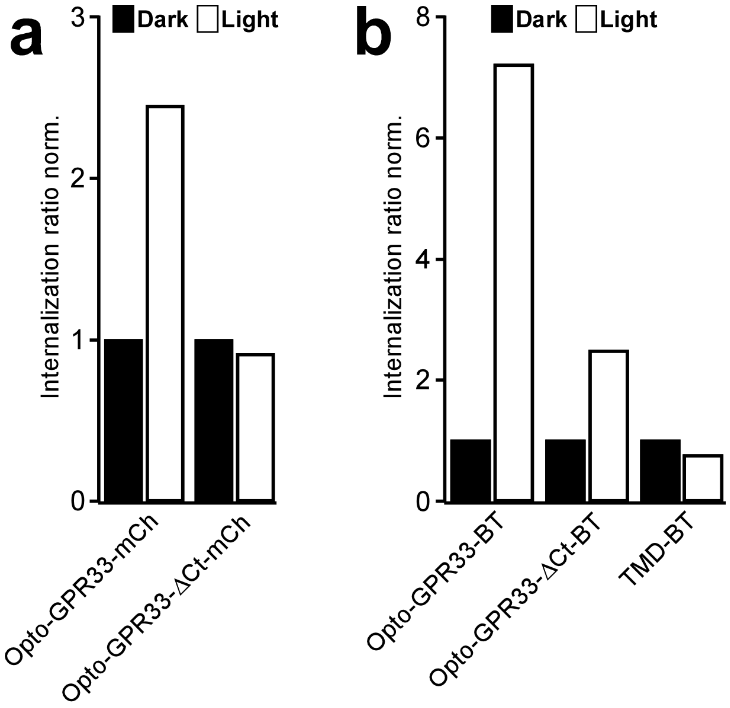

Supplementary Figure 8. Analysis of images shown in Supplementary Fig. 7. Internalization ratios are defined as the number of cells with internalized receptors divided by the number of cells without internalized receptors (see **Methods**). Mean ratios (n=3-6 wells) are shown.

Supplementary Table 1. Reference GPCRs.

Receptor	Species	Predom. G_α coupling[a)]	Expression vector	Source[c)]
β2 adrenergic receptor (β2AR)	Human	G_αs	pcDNA3	Robert Lefkowitz (Duke Univ.) *via* Addgene, (14697)
α1 adrenergic receptor (α1AR)	Rat	G_αq	pCMV5	Robert Lefkowitz (Duke Univ.) *via* Addgene, (45760)
Free fatty acid receptor 3 (FFR3)	Human	$G_\alpha$io and G_αq	pcDNA3	Graeme Milligan (Univ. of Glasgow)
Adenosine A2A receptor (A2AR)	Human	G_αs	N/A[b)]	N/A
Dopamine receptor D1 (D1R)	Human	G_αs	pcDNA3.1	MGC (202856822)
Dopamine receptor D2 (D2R)	Human	$G_\alpha$io	pcDNA3.1	MGC (202830262)
Muscarinic acetylcholine receptor M1 (M1R)	Human	G_αq and G_αs	pcDNA3.1	Klaus Groschner (Med. Univ. of Graz)
Muscarinic acetylcholine receptor M2 (M2R)	Human	$G_\alpha$io	pcDNA3.1	MGC (211689788)
Muscarinic acetylcholine receptor M3 (M3R)	Human	G_αq	N/A [b)]	N/A

[a)] G_α-coupling as retrieved from literature.

[b)] N/A: HEK293 cells endogenously express adenosine receptors (A2A/BR) and muscarinic acetylcholine receptors (M3R) [2].

[c)] MGC: Mammalian Gene Collection clones obtained from GE Healthcare Dharmacon.

Supplementary Table 2. Ligands used to activate reference GPCRs.

Receptor(s)	Ligand (agonists unless noted otherwise)	Stock solvent	Stock concentration	Final concentration
α1AR<br>β2AR	Norepinephrine	DMSO	19 mM	10 μM [3]
FFR3	Propionate	water	2 M	10 mM[4]
A2AR	5′-(N-ethylcarboxamido) adenosine	DMSO	80 mM	100 μM[5]
D1R<br>D2R	Dopamine	DMSO	100 mM	100 μM[6,7]
M2R<br>M3R	Muscarine	DMSO	24 mM	100 μM[8,9]
M1R	VU357017 (positive, allosteric modulator)	DMSO	5 mM	25 μM[10]

Supplementary Table 3. Receptors listed by IUPHAR and included in this study. See **Methods** for gene selection and curation.

Gene name	SwissProt/Uniprot identifier
GPR1	P46091
GPR3	P46089
GPR4	P46093
GPR6	P46095
GPR12	P47775
GPR15	P49685
GPR17	Q13304
GPR18	Q14330
GPR19	Q15760[a)]
GPR20	Q99678
GPR21	Q99679
GPR22	Q99680
GPR25	O00155
GPR26	Q8NDV2
GPR27	Q9NS67
GPR31	O00270
GPR32	O75388
GPR33	Q49SQ1
GPR34	Q9UPC5
GPR35	Q9HC97
GPR37	O15354
GPR37L1	O60883
GPR39	O43194
GPR42	O15529[a)]
GPR45	Q9Y5Y3
GPR50	Q13585
GPR52	Q9Y2T5
GPR55	Q9Y2T6
GPR61	Q9BZJ8
GPR62	Q9BZJ7
GPR63	Q9BZJ6
GPR65	Q8IYL9
GPR68	Q15743
GPR75	O95800
GPR78	Q96P69
GPR82	Q96P67
GPR83	Q9NYM4
GPR84	Q9NQS5

GPR85	P60893
GPR87	Q9BY21
GPR88	Q9GZN0
GPR119	Q8TDV5
GPR120	Q5NUL3
GPR132	Q9UNW8
GPR135	Q8IZ08
GPR139	Q6DWJ6
GPR141	Q7Z602
GPR142	Q7Z601
GPR146	Q96CH1
GPR148	Q8TDV2
GPR149	Q86SP6
GPR150	Q8NGU9
GPR151	Q8TDV0
GPR152	Q8TDT2
GPR153	Q6NV75
GPR160	Q9UJ42
GPR161	Q8N6U8
GPR162	Q16538
GPR171	O14626
GPR173	Q9NS66
GPR174	Q9BXC1
GPR176	Q14439
GPR182	O15218
GPR183	P32249
[a)] Since the start of this study, two SwissProt/Uniprot sequences changed in the repository at a single amino acid site each.	

Supplementary Table 4. Design of receiver gene and inserts (here, for GPR1 as an example) for high-throughput cloning. Color coding of receiver gene corresponds to that of **Supplementary Fig. 1** and is further described below. Double-stranded inserts (green) can be obtained either by annealing oligonucleotides with sticky ends (e.g. 5’-CGTCACGGGGTTCAAGTGGAAGAAAACAGTC-3’ and 5’-TGAGGACTGTTTTCTTCCACTTGAACCCCGT-3’ for GPR1_insert_1) or from a vector with orthogonal antibiotics resistance (e.g. 5’-GCTCTTCACGCGCT…CAAACAAGAAGAGC-3’ for GPR1_insert_4).

>receiver_gene

ACCATGAAAACGATCATCGCCCTGAGCTACATCTTCTGCCTGGTATTCGCCATGTACACCGATATAGAGATGAACAGGCTGGGAAAGGATAGCCTCATGAACGGGACCGAGGGCCCAAACTTCTACGTGCCTTTCTCCAACA

AGACGGGCGTGGTGCGCAGCCCCTTCGAGGCCCCGCAGTACTACCTGGCGGAGCCATGGCAGTTCTCCATGCTGGCCGCCTACATGTTCCTGCTGATCATGCTTGGCTTCCCCATCAACTTCCTCACGCTGTACGTCAGAGA

CCCAGTCCAGCAACCGGTCAAGAAGCTGGGTCTCCCTCAACTACATCCTGCTCAACCTGGCCGTGGCCGACCTCTTCATGGTGTTCGGGGGCTTCACCACCACCCTCTACACCTCTCTGCACGGGTACTTCGTCTTTGGGCC

CACGGGCTGCAACCTGGAGGGCTTCTTTGCCACCTTGGGCGGTGAAATTGCACTGTGGTCCTTGGTGGTCCTGGCCATCGAGCGGTACGTGGTGGTGTGAGACGGCAAGCCCATGCTAAGCGAGCAACTTCCCGTCTCGGCC

ATCATGGGCGTCGCCTTCACCTGGGTCATGGCTCTGGCCTGTGCCGCGCCCCCCTCGTCGGCTGGTCCAGGTACATCCCGGAGGGCATGCAGTGCTCGTGCGGGATTGACTACTACACGCCCCACGAGGAAACCAACAATG

AGTCGTTCGTCATCTACATGTTCGTGGTCCACTTCATCATCCCCCTGATTGTCATATTCTTCTGCTACGGACAGTCTTCGCTGGTGTTGATATCCACCGTCAAGGGAAGACAGCGCATGGTGATCATCATGGTCATCGCTTT

CCTAATCTGCTGGCTGCCCTACGCTGGGGTGGCGTTCTACATCTTCACCCATCAGGGCTCTGACTTTGGCCCCATCTTCATGACCATCCCGGCTTTCTTTGCCAAGACTTCTGCCGTCTACAACCCCGTCATCTACATCATG

ATGAACAAGCAGTTCCGNAGAAGAGCACTGCATGGTGCGGCCGCCACCACTCTCGCTCTTCTACAGAAACCAGCCAAGTGGCGCCTGCCTAA

>GPR1_insert_1 (ICL1)

CGTCACGGGGTTCAAGTGGAAGAAAACAGTCCTCA

>GPR1_insert_2 (ICL2)

GGTGATCCATCCTGTCTTATCTCATCGGCATCGAACCCTCAAGAACGCCA

>GPR1_insert_3 (ICL3)

CGGATGTCTCATCTTCAAGGTGAAGAAGCGAAGCATCCTGATCTCCAGTAGGCATTTCCGCA

>GPR1_insert_4 (C-terminus)

CGCGCTCGCTTCCGGTCCTCAGTTGCTGAGATACTCAAGTACACACTGTGGGAAGTCAGCTGTTCTGGCACAGTGAGTGAACAGCTCAGGAACTCAGAAACCAAGAATCTGTGTCTCCTGGAAACAGCTCAAACA

>final_assembled_gene_Opto-GPR1

ACCATGAAAACGATCATCGCCCTGAGCTACATCTTCTGCCTGGTATTCGCCATGTACACCGATATAGAGATGAACAGGCTGGGAAAGGATAGCCTCATGAACGGGACCGAGGGCCCAAACTTCTACGTGCCTTTCTCCAACA

AGACGGGCGTGGTGCGCAGCCCCTTCGAGGCCCCGCAGTACTACCTGGCGGAGCCATGGCAGTTCTCCATGCTGGCCGCCTACATGTTCCTGCTGATCATGCTTGGCTTCCCCATCAACTTCCTCACGCTGTACGTCACGGG

GTTCAAGTGGAAGAAAACAGTCCTCAACTACATCCTGCTCAACCTGGCCGTGGCCGACCTCTTCATGGTGTTCGGGGGCTTCACCACCACCCTCTACACCTCTCTGCACGGGTACTTCGTCTTTGGGCCCACGGGCTGCAAC

CTGGAGGGCTTCTTTGCCACCTTGGGCGGTGAAATTGCACTGTGGTCCTTGGTGGTCCTGGCCATCGAGCGGTACGTGGTGGTGATCCATCCTGTCTTATCTCATCGGCATCGAACCCTCAAGAACGCCATCATGGGCGTCG

CCTTCACCTGGGTCATGGCTCTGGCCTGTGCCGCGCCCCCCCTCGTCGGCTGGTCCAGGTACATCCCGGAGGGCATGCAGTGCTCGTGCGGGATTGACTACTACACGCCCCACGAGGAAACCAACAATGAGTCGTTCGTCAT

CTACATGTTCGTGGTCCACTTCATCATCCCCCTGATTGTCATATTCTTCTGCTACGGATGTCTCATCTTCAAGGTGAAGAAGCGAAGCATCCTGATCTCCAGTAGGCATTTCCGCATGGTGATCATCATGGTCATCGCTTTC

CTAATCTGCTGGCTGCCCTACGCTGGGGTGGCGTTCTACATCTTCACCCATCAGGGCTCTGACTTTGGCCCCATCTTCATGACCATCCCGGCTTTCTTTGCCAAGACTTCTGCCGTCTACAACCCCGTCATCTACATCATGA

TGAACAAGCAGTTCCGCGCTCGCTTCCGGTCCTCAGTTGCTGAGATACTCAAGTACACACTGTGGGAAGTCAGCTGTTCTGGCACAGTGAGTGAACAGCTCAGGAACTCAGAAACCAAGAATCTGTGTCTCCTGGAAACAGC

TCAAACAGAAACCAGCCAAGTGGCGCCTGCCTAA

Color code:

Kozak sequence and start codon

Signal peptide and VSV-G epitope (not shown in **Supplementary Fig. 1**)

Rhodopsin coding sequence

Insert coding sequence

Type IIs restriction sites: BsaI (ICL1), BsmBI (ICL2), BpiI/BbsI (ICL3), LguI/SapI (C-terminus)

Overhangs (not shown in **Supplementary Fig. 1**)

Additional restrictions sites: AgeI, BlpI, EcoRV, NotI (not shown in **Supplementary Fig. 1**)

N: C or T (not shown in **Supplementary Fig. 1**)

Stop codon

Supplementary Table 5. Nucleotide sequence of modified expression vector. See **Supplementary Fig. 2** for description of vector. Removed recognition sites for BsaI, BbsI and SapI restriction enzymes are highlighted in gray (two sites), dark green (one site) and light blue (three sites), resp. Red highlights point to introduced substitutions. The modified vector is available through Addgene.org.

```
GACGGATCGGGAGATCTCCCGATCCCCTATGGTGCACTCTCAGTACAATCTGCTCTGATGCCGCATAGTTAAGCCAGTATCTGCTCCCTGCTTGTG
TGTTGGAGGTCGCTGAGTAGTGCGCGAGCAAAATTTAAGCTACAACAAGGCAAGGCTTGACCGACAATTGCATGAAGAATCTGCTTAGGGTTAGGC
GTTTTGCGCTGCTTCGCGATGTACGGGCCAGATATACGCGTTGACATTGATTATTGACTAGTTATTAATAGTAATCAATTACGGGGTCATTAGTTC
ATAGCCCATATATGGAGTTCCGCGTTACATAACTTACGGTAAATGGCCCGCCTGGCTGACCGCCCAACGACCCCCGCCCATTGACGTCAATAATGA
CGTATGTTCCCATAGTAACGCCAATAGGGACTTTCCATTGACGTCAATGGGTGGAGTATTTACGGTAAACTGCCCACTTGGCAGTACATCAAGTGT
ATCATATGCCAAGTACGCCCCCTATTGACGTCAATGACGGTAAATGGCCCGCCTGGCATTATGCCCAGTACATGACCTTATGGGACTTTCCTACTT
GGCAGTACATCTACGTATTAGTCATCGCTATTACCATGGTGATGCGGTTTTGGCAGTACATCAATGGGCGTGGATAGCGGTTTGACTCACGGGGAT
TTCCAAGTCTCCACCCCATTGACGTCAATGGGAGTTTGTTTTGGCACCAAAATCAACGGGACTTTCCAAAATGTCGTAACAACTCCGCCCCATTGA
CGCAAATGGGCGGTAGGCGTGTACGGTGGGAGGTCTATATAAGCAGAGCTCTCTGGCTAACTAGAGAACCCACTGCTTACTGGCTTATCGAAATTA
ATACGACTCACTATAGGGAGACACAAGCTGGCTAGCGTTTAAACGGGCCCTCTAGACTCGAGCGGCCGCCACTGTGCTGGATATCTGCAGAATTCC
ACCACACTGGACTAGTGGATCCGAGCTCGGTACCAAGCTTAAGTTTAAACCGCTGATCAGCCTCGACTGTGCCTTCTAGTTGCCAGCCATCTGTTG
TTTGCCCCTCCCCCGTGCCTTCCTTGACCCTGGAAGGTGCCACTCCCACTGTCCTTTCCTAATAAAATGAGGAAATTGCATCGCATTGTCTGAGTA
GGTGTCATTCTATTCTGGGGGGTGGGGTGGGGCAGGACAGCAAGGGGGAGGATTGGGAAGAGAATAGCAGGCATGCTGGGGATGCGGTGGGCTCTA
TGGCTTCTGAGGCGGAAAGAACCAGCTGGGGCTCTAGGGGGTATCCCCACGCGCCCTGTAGCGGCGCATTAAGCGCGGCGGGTGTGGTGGTTACGC
GCAGCGTGACCGCTACACTTGCCAGCGCCCTAGCGCCCGCTCCTTTCGCTTTCTTCCCTTCCTTTCTCGCCACGTTCGCCGGCTTTCCCCGTCAAG
CTCTAAATCGGGGGCTCCCTTTAGGGTTCCGATTTAGTGCTTTACGGCACCTCGACCCCAAAAAACTTGATTAGGGTGATGGTTCACGTAGTGGGC
CATCGCCCTGATAGACGGTTTTTCGCCCTTTGACGTTGGAGTCCACGTTCTTTAATAGTGGACTCTTGTTCCAAACTGGAACAACACTCAACCCTA
TCTCGGTCTATTCTTTTGATTTATAAGGGATTTTGCCGATTTCGGCCTATTGGTTAAAAAATGAGCTGATTTAACAAAAATTTAACGCGAATTAAT
TCTGTGGAATGTGTGTCAGTTAGGGTGTGGAAAGTCCCCAGGCTCCCCAGCAGGCAGAAGTATGCAAAGCATGCATCTCAATTAGTCAGCAACCAG
GTGTGGAAAGTCCCCAGGCTCCCCAGCAGGCAGAAGTATGCAAAGCATGCATCTCAATTAGTCAGCAACCATAGTCCCGCCCCTAACTCCGCCCAT
CCCGCCCCTAACTCCGCCCAGTTCCGCCCATTCTCCGCCCCATGGCTGACTAATTTTTTTTATTTATGCAGAGGCCGAGGCCGCCTCTGCCTCTGA
GCTATTCCAGAAGTAGTGAGGAGGCTTTTTTGGAGGCCTAGGCTTTTGCAAAAAGCTCCCGGGAGCTTGTATATCCATTTTCGGATCTGATCAAGA
GACAGGATGAGGATCGTTTCGCATGATTGAACAAGATGGATTGCACGCAGGTTCTCCGGCCGCTTGGGTGGAGAGGCTATTCGGCTATGACTGGGC
ACAACAGACAATCGGCTGCTCTGATGCCGCCGTGTTCCGGCTGTCAGCGCAGGGGCGCCCGGTTCTTTTTGTCAAGACCGACCTGTCCGGTGCCCT
GAATGAACTGCAGGACGAGGCAGCGCGGCTATCGTGGCTGGCCACGACGGGCGTTCCTTGCGCAGCTGTGCTCGACGTTGTCACTGAAGCGGGAAG
GGACTGGCTGCTATTGGGCGAAGTGCCGGGGCAGGATCTCCTGTCATCTCACCTTGCTCCTGCCGAGAAAGTATCCATCATGGCTGATGCAATGCG
GCGGCTGCATACGCTTGATCCGGCTACCTGCCCATTCGACCACCAAGCGAAACATCGCATCGAGCGAGCACGTACTCGGATGGAAGCCGGTCTTGT
CGATCAGGATGATCTGGACGAAGAACATCAGGGGCTCGCGCCAGCCGAACTGTTCGCCAGGCTCAAGGCGCGCATGCCCGACGGCGAGGATCTCGT
CGTGACCCATGGCGATGCCTGCTTGCCGAATATCATGGTGGAAAATGGCCGCTTTTCTGGATTCATCGACTGTGGCCGGCTGGGTGTGGCGGACCG
CTATCAGGACATAGCGTTGGCTACCCGTGATATTGCTGAAGAACTTGGCGGCGAATGGGCTGACCGCTTCCTCGTGCTTTACGGTATCGCCGCTCC
```

CGATTCGCAGCGCATCGCCTTCTATCGCCTTCTTGACGAGTTCTTCTGAGCGGGACTCTGGGGTTCGAAATGACCGACCAAGCGACGCCCAACCTG

CCATCACGAGATTTCGATTCCACCGCCGCCTTCTATGAAAGGTTGGGCTTCGGAATCGTTTTCCGGGACGCCGGCTGGATGATCCTCCAGCGCGGG

GATCTCATGCTGGAGTTCTTCGCCCACCCCAACTTGTTTATTGCAGCTTATAATGGTTACAAATAAAGCAATAGCATCACAAATTTCACAAATAAA

GCATTTTTTTCACTGCATTCTAGTTGTGGTTTGTCCAAACTCATCAATGTATCTTATCATGTCTGTATACCGTCGACCTCTAGCTAGAGCTTGGCG

TAATCATGGTCATAGCTGTTTCCTGTGTGAAATTGTTATCCGCTCACAATTCCACACAACATACGAGCCGGAAGCATAAAGTGTAAAGCCTGGGGT

GCCTAATGAGTGAGCTAACTCACATTAATTGCGTTGCGCTCACTGCCCGCTTTCCAGTCGGGAAACCTGTCGTGCCAGCTGCATTAATGAATCGGC

CAACGCGCGGGGAGAGGCGGTTTGCGTATTGGGCGCTCTTGCGCTTCCTCGCTCACTGACTCGCTGCGCTCGGTCGTTCGGCTGCGGCGAGCGGTA

TCAGCTCACTCAAAGGCGGTAATACGGTTATCCACAGAATCAGGGGATAACGCAGGAAAGAACATGTGAGCAAAAGGCCAGCAAAAGGCCAGGAAC

CGTAAAAAGGCCGCGTTGCTGGCGTTTTTCCATAGGCTCCGCCCCCCTGACGAGCATCACAAAAATCGACGCTCAAGTCAGAGGTGGCGAAACCCG

ACAGGACTATAAAGATACCAGGCGTTTCCCCCTGGAAGCTCCCTCGTGCGCTCTCCTGTTCCGACCCTGCCGCTTACCGGATACCTGTCCGCCTTT

CTCCCTTCGGGAAGCGTGGCGCTTTCTCATAGCTCACGCTGTAGGTATCTCAGTTCGGTGTAGGTCGTTCGCTCCAAGCTGGGCTGTGTGCACGAA

CCCCCCGTTCAGCCCGACCGCTGCGCCTTATCCGGTAACTATCGTCTTGAGTCCAACCCGGTAAGACACGACTTATCGCCACTGGCAGCAGCCACT

GGTAACAGGATTAGCAGAGCGAGGTATGTAGGCGGTGCTACAGAGTTCTTGAAGTGGTGGCCTAACTACGGCTACACTAGAAGAACAGTATTTGGT

ATCTGCGCTCTGCTGAAGCCAGTTACCTTCGGAAAAAGAGTTGGTAGCTCTTGATCCGGCAAACAAACCACCGCTGGTAGCGGTTTTTTTGTTTGC

AAGCAGCAGATTACGCGCAGAAAAAAAGGATCTCAAGAAGATCCTTTGATCTTTTCTACGGGGTCTGACGCTCAGTGGAACGAAAACTCACGTTAA

GGGATTTTGGTCATGAGATTATCAAAAAGGATCTTCACCTAGATCCTTTTAAATTAAAAATGAAGTTTTAAATCAATCTAAAGTATATATGAGTAA

ACTTGGTCTGACAGTTACCAATGCTTAATCAGTGAGGCACCTATCTCAGCGATCTGTCTATTTCGTTCATCCATAGTTGCCTGACTCCCCGTCGTG

TAGATAACTACGATACGGGAGGGCTTACCATCTGGCCCCAGTGCTGCAATGATACCGCGGGACCCACGCTCACCGGCTCCAGATTTATCAGCAATA

AACCAGCCAGCCGGAAGGGCCGAGCGCAGAAGTGGTCCTGCAACTTTATCCGCCTCCATCCAGTCTATTAATTGTTGCCGGGAAGCTAGAGTAAGT

AGTTCGCCAGTTAATAGTTTGCGCAACGTTGTTGCCATTGCTACAGGCATCGTGGTGTCACGCTCGTCGTTTGGTATGGCTTCATTCAGCTCCGGT

TCCCAACGATCAAGGCGAGTTACATGATCCCCCATGTTGTGCAAAAAAGCGGTTAGCTCCTTCGGTCCTCCGATCGTTGTCAGAAGTAAGTTGGCC

GCAGTGTTATCACTCATGGTTATGGCAGCACTGCATAATTCTCTTACTGTCATGCCATCCGTAAGATGCTTTTCTGTGACTGGTGAGTACTCAACC

AAGTCATTCTGAGAATAGTGTATGCGGCGACCGAGTTGCTCTTGCCCGGCGTCAATACGGGATAATACCGCGCCACATAGCAGAACTTTAAAAGTG

CTCATCATTGGAAAACGTTCTTCGGGGCGAAAACTCTCAAGGATCTTACCGCTGTTGAGATCCAGTTCGATGTAACCCACTCGTGCACCCAACTGA

TCTTCAGCATCTTTTACTTTCACCAGCGTTTCTGGGTGAGCAAAAACAGGAAGGCAAAATGCCGCAAAAAAGGGAATAAGGGCGACACGGAAATGT

TGAATACTCATACTCTTCCTTTTTCAATATTATTGAAGCATTTATCAGGGTTATTGTCTCATGAGCGGATACATATTTGAATGTATTTAGAAAAAT

AAACAAATAGGGGTTCCGCGCACATTTCCCCGAAAAGTGCCACCTGACGTC

Supplementary Table 6. Evaluation of parameters for high-throughput cloning technique.

Condition	Cycles	Additional digest	Colony forming units	Positive clones
Receptor 1: GPR33				
1	33	Yes	600	10/10
2	33	No	400	10/10
3	60	Yes	100	10/10
Receptor 2: α1AR				
1	33	Yes	450	7/10
2	33	No	1500	3/10
3	60	Yes	300	9/10

Supplementary Table 7. Tabulated results for data shown in Fig. 3 (mean ± s.e.m., n=6-9, 3-4 independent experiments).

	CRE		SRE		SRE.L		CRE+stim.	
**Gene name**	**Dark**	**Light**	**Dark**	**Light**	**Dark**	**Light**	**Dark**	**Light**
Opto-GPR1	1.52 ± 0.42	8.94 ± 3.52	0.52 ± 0.17	0.61 ± 0.22	0.74 ± 0.25	0.57 ± 0.20	1.39 ± 0.12	1.45 ± 0.26
Opto-GPR3	0.45 ± 0.16	0.33 ± 0.07	0.68 ± 0.23	3.63 ± 2.95	0.63 ± 0.19	0.44 ± 0.17	1.03 ± 0.13	0.92 ± 0.18
Opto-GPR4	0.47 ± 0.18	0.30 ± 0.07	1.61 ± 1.00	1.84 ± 1.36	0.67 ± 0.25	0.50 ± 0.38	0.60 ± 0.12	0.36 ± 0.11
Opto-GPR6	0.49 ± 0.26	0.25 ± 0.08	1.26 ± 0.84	1.09 ± 0.46	0.69 ± 0.21	0.51 ± 0.20	1.02 ± 0.08	0.83 ± 0.2
Opto-GPR12	0.19 ± 0.08	0.20 ± 0.06	1.17 ± 0.58	0.76 ± 0.30	0.80 ± 0.28	0.58 ± 0.27	1.14 ± 0.19	0.95 ± 0.15
Opto-GPR15	0.26 ± 0.14	0.21 ± 0.07	0.54 ± 0.29	0.65 ± 0.31	0.77 ± 0.25	0.73 ± 0.32	1.02 ± 0.13	0.77 ± 0.13
Opto-GPR17	0.32 ± 0.10	0.27 ± 0.08	0.49 ± 0.25	0.60 ± 0.24	0.87 ± 0.34	0.54 ± 0.17	1.00 ± 0.08	0.83 ± 0.15
Opto-GPR18	0.19 ± 0.06	0.22 ± 0.06	0.32 ± 0.07	0.80 ± 0.49	0.87 ± 0.28	0.56 ± 0.21	1.00 ± 0.12	1.14 ± 0.33
Opto-GPR19	0.21 ± 0.08	0.24 ± 0.09	0.46 ± 0.23	0.55 ± 0.25	0.73 ± 0.22	0.63 ± 0.28	1.03 ± 0.14	0.90 ± 0.21
Opto-GPR20	0.23 ± 0.03	0.49 ± 0.20	0.49 ± 0.20	0.86 ± 0.29	0.59 ± 0.15	0.64 ± 0.24	0.88 ± 0.10	0.74 ± 0.19
Opto-GPR21	0.36 ± 0.07	10.36 ± 5.73	1.66 ± 1.36	0.57 ± 0.22	0.62 ± 0.19	0.64 ± 0.22	0.86 ± 0.08	0.99 ± 0.31
Opto-GPR22	0.27 ± 0.09	0.41 ± 0.12	1.41 ± 0.74	1.27 ± 0.59	0.69 ± 0.18	0.86 ± 0.31	1.05 ± 0.29	2.45 ± 1.34
Opto-GPR25	0.29 ± 0.08	0.40 ± 0.08	0.97 ± 0.61	1.44 ± 1.03	0.64 ± 0.22	0.45 ± 0.15	1.29 ± 0.17	0.98 ± 0.19
Opto-GPR26	0.36 ± 0.10	0.41 ± 0.09	1.20 ± 0.84	0.54 ± 0.14	0.53 ± 0.17	0.44 ± 0.15	1.15 ± 0.11	0.86 ± 0.15
Opto-GPR27	0.32 ± 0.10	0.23 ± 0.04	0.56 ± 0.20	0.59 ± 0.21	0.58 ± 0.17	0.41 ± 0.15	1.01 ± 0.06	0.89 ± 0.14
Opto-GPR31	0.27 ± 0.11	0.57 ± 0.27	0.56 ± 0.19	0.79 ± 0.38	0.57 ± 0.21	0.51 ± 0.17	1.02 ± 0.10	0.80 ± 0.15
Opto-GPR32	0.21 ± 0.07	4.89 ± 2.32	0.81 ± 0.38	0.67 ± 0.36	0.54 ± 0.16	0.44 ± 0.13	1.04 ± 0.09	0.66 ± 0.15
Opto-GPR33	0.31 ± 0.14	0.46 ± 0.10	0.30 ± 0.07	2.45 ± 1.33	0.62 ± 0.16	30.56 ± 10.23	0.97 ± 0.09	0.65 ± 0.13
Opto-GPR34	0.30 ± 0.11	0.30 ± 0.09	0.49 ± 0.16	0.50 ± 0.14	0.61 ± 0.27	0.65 ± 0.16	0.95 ± 0.09	0.81 ± 0.09
Opto-GPR35	0.32 ± 0.11	0.22 ± 0.04	0.33 ± 0.08	0.47 ± 0.16	0.63 ± 0.16	0.64 ± 0.21	1.03 ± 0.10	0.74 ± 0.12
Opto-GPR37	0.30 ± 0.08	0.25 ± 0.07	0.50 ± 0.11	0.41 ± 0.13	0.53 ± 0.12	0.47 ± 0.16	0.91 ± 0.08	0.91 ± 0.15
Opto-GPR37L1	0.25 ± 0.08	0.31 ± 0.09	0.33 ± 0.11	0.33 ± 0.08	0.49 ± 0.13	0.50 ± 0.19	0.89 ± 0.11	1.06 ± 0.20
Opto-GPR39	0.39 ± 0.19	0.51 ± 0.21	0.67 ± 0.21	0.35 ± 0.10	0.52 ± 0.15	0.63 ± 0.19	0.87 ± 0.05	0.92 ± 0.21
Opto-GPR42	0.30 ± 0.14	1.05 ± 0.68	0.59 ± 0.21	0.49 ± 0.18	0.61 ± 0.15	0.60 ± 0.20	0.88 ± 0.05	1.22 ± 0.46
Opto-GPR45	0.43 ± 0.16	0.56 ± 0.12	1.38 ± 0.60	0.71 ± 0.24	0.53 ± 0.13	0.48 ± 0.15	1.16 ± 0.18	0.85 ± 0.07
Opto-GPR50	0.40 ± 0.16	0.43 ± 0.06	0.81 ± 0.20	0.86 ± 0.28	0.51 ± 0.15	0.34 ± 0.12	1.07 ± 0.19	1.07 ± 0.13
Opto-GPR52	0.26 ± 0.09	0.30 ± 0.07	0.93 ± 0.49	0.65 ± 0.24	0.54 ± 0.15	0.45 ± 0.17	0.99 ± 0.07	0.88 ± 0.11
Opto-GPR55	0.32 ± 0.12	0.46 ± 0.09	0.74 ± 0.20	0.66 ± 0.20	0.50 ± 0.15	0.40 ± 0.10	1.12 ± 0.10	0.75 ± 0.15
Opto-GPR61	0.62 ± 0.17	6.11 ± 3.10	0.58 ± 0.20	0.66 ± 0.29	0.51 ± 0.15	0.62 ± 0.18	1.07 ± 0.12	0.82 ± 0.12
Opto-GPR62	0.32 ± 0.09	0.51 ± 0.14	0.57 ± 0.22	0.77 ± 0.22	0.55 ± 0.16	0.60 ± 0.11	0.89 ± 0.04	0.83 ± 0.15
Opto-GPR63	0.37 ± 0.11	0.51 ± 0.13	0.42 ± 0.12	0.44 ± 0.16	0.53 ± 0.18	0.78 ± 0.16	1.16 ± 0.28	0.74 ± 0.15
Opto-GPR65	0.39 ± 0.15	0.53 ± 0.21	0.59 ± 0.19	0.67 ± 0.26	0.54 ± 0.14	0.58 ± 0.16	1.02 ± 0.05	0.80 ± 0.08
Opto-GPR68	0.81 ± 0.45	0.78 ± 0.31	0.97 ± 0.27	2.61 ± 0.76	0.52 ± 0.15	0.58 ± 0.17	0.84 ± 0.05	1.02 ± 0.09
Opto-GPR75	0.33 ± 0.14	0.30 ± 0.08	0.99 ± 0.45	1.28 ± 0.93	0.61 ± 0.18	0.62 ± 0.15	0.89 ± 0.09	0.86 ± 0.13
Opto-GPR78	0.29 ± 0.05	0.65 ± 0.24	1.00 ± 0.34	5.05 ± 2.87	0.54 ± 0.12	35.1 ± 11.47	0.96 ± 0.13	1.38 ± 0.32
Opto-GPR82	0.28 ± 0.07	0.50 ± 0.14	0.59 ± 0.19	0.54 ± 0.21	0.68 ± 0.17	0.71 ± 0.15	0.93 ± 0.14	1.31 ± 0.43
Opto-GPR83	0.81 ± 0.18	0.67 ± 0.14	0.95 ± 0.19	0.86 ± 0.25	0.56 ± 0.22	0.51 ± 0.16	1.23 ± 0.26	1.09 ± 0.17

Opto-GPR84	0.47 ± 0.09	1.02 ± 0.27	0.75 ± 0.23	0.67 ± 0.19	0.49 ± 0.16	0.45 ± 0.13	1.03 ± 0.14	1.04 ± 0.12
Opto-GPR85	0.55 ± 0.15	1.10 ± 0.38	0.54 ± 0.16	0.43 ± 0.10	0.39 ± 0.10	0.49 ± 0.15	0.97 ± 0.13	0.72 ± 0.15
Opto-GPR87	0.44 ± 0.09	0.53 ± 0.09	0.59 ± 0.18	0.66 ± 0.18	0.44 ± 0.12	0.45 ± 0.13	1.06 ± 0.05	1.00 ± 0.24
Opto-GPR88	0.52 ± 0.08	0.44 ± 0.04	0.55 ± 0.17	1.88 ± 0.92	0.48 ± 0.15	0.55 ± 0.19	1.04 ± 0.06	1.02 ± 0.12
Opto-GPR119	1.27 ± 0.45	0.87 ± 0.23	0.64 ± 0.17	0.67 ± 0.14	0.47 ± 0.09	0.52 ± 0.10	1.06 ± 0.14	0.90 ± 0.10
Opto-GPR120	0.69 ± 0.18	0.46 ± 0.20	1.78 ± 0.79	1.59 ± 1.02	1.22 ± 0.54	0.80 ± 0.64	0.65 ± 0.24	0.77 ± 0.19
Opto-GPR132	1.33 ± 0.43	0.66 ± 0.20	0.57 ± 0.18	0.55 ± 0.15	0.50 ± 0.11	0.69 ± 0.17	0.96 ± 0.09	0.89 ± 0.07
Opto-GPR135	0.57 ± 0.12	6.78 ± 3.09	0.64 ± 0.09	1.11 ± 0.45	0.58 ± 0.14	0.55 ± 0.14	1.07 ± 0.10	1.09 ± 0.23
Opto-GPR139	0.42 ± 0.09	0.43 ± 0.11	0.41 ± 0.08	0.53 ± 0.11	0.46 ± 0.11	0.55 ± 0.12	0.80 ± 0.03	1.18 ± 0.41
Opto-GPR141	0.55 ± 0.13	0.37 ± 0.08	0.58 ± 0.15	0.45 ± 0.12	0.63 ± 0.16	0.68 ± 0.20	0.93 ± 0.08	1.28 ± 0.41
Opto-GPR142	0.33 ± 0.12	0.30 ± 0.05	0.67 ± 0.29	0.46 ± 0.14	0.70 ± 0.17	0.91 ± 0.19	0.76 ± 0.04	0.95 ± 0.23
Opto-GPR146	0.65 ± 0.15	1.06 ± 0.43	0.96 ± 0.21	1.62 ± 0.89	0.60 ± 0.17	0.58 ± 0.12	1.50 ± 0.45	1.09 ± 0.24
Opto-GPR148	0.55 ± 0.13	0.78 ± 0.39	1.05 ± 0.29	0.68 ± 0.25	0.81 ± 0.4	0.58 ± 0.16	1.09 ± 0.07	0.95 ± 0.13
Opto-GPR149	0.46 ± 0.10	1.01 ± 0.39	0.88 ± 0.3	0.69 ± 0.17	0.66 ± 0.28	0.54 ± 0.18	1.21 ± 0.15	0.91 ± 0.19
Opto-GPR150	0.74 ± 0.26	1.05 ± 0.32	1.43 ± 0.31	1.26 ± 0.33	0.48 ± 0.12	0.70 ± 0.23	1.15 ± 0.10	0.73 ± 0.14
Opto-GPR151	0.52 ± 0.09	0.75 ± 0.18	0.65 ± 0.17	1.17 ± 0.25	0.87 ± 0.49	0.49 ± 0.12	0.83 ± 0.06	0.90 ± 0.09
Opto-GPR152	0.42 ± 0.08	0.48 ± 0.10	0.56 ± 0.17	0.74 ± 0.26	0.52 ± 0.10	0.68 ± 0.16	0.87 ± 0.13	0.88 ± 0.17
Opto-GPR153	0.67 ± 0.15	0.67 ± 0.09	0.66 ± 0.21	0.69 ± 0.18	0.54 ± 0.12	0.75 ± 0.19	0.93 ± 0.10	1.10 ± 0.16
Opto-GPR160	0.44 ± 0.04	0.58 ± 0.15	0.67 ± 0.26	0.86 ± 0.51	0.87 ± 0.13	0.83 ± 0.13	1.13 ± 0.12	1.02 ± 0.15
Opto-GPR161	0.57 ± 0.08	0.67 ± 0.13	0.66 ± 0.21	0.65 ± 0.21	0.70 ± 0.18	0.74 ± 0.20	0.88 ± 0.11	1.04 ± 0.25
Opto-GPR162	0.39 ± 0.10	0.57 ± 0.08	0.61 ± 0.13	0.88 ± 0.34	0.73 ± 0.14	0.95 ± 0.24	0.98 ± 0.06	1.68 ± 0.79
Opto-GPR171	0.54 ± 0.09	0.54 ± 0.11	0.70 ± 0.10	0.73 ± 0.21	0.63 ± 0.12	0.74 ± 0.13	1.08 ± 0.06	2.19 ± 1.20
Opto-GPR173	0.37 ± 0.09	0.38 ± 0.09	0.59 ± 0.14	0.57 ± 0.13	1.98 ± 1.18	0.80 ± 0.11	1.1 ± 0.06	1.46 ± 0.27
Opto-GPR174	1.01 ± 0.23	0.87 ± 0.19	1.28 ± 0.69	0.68 ± 0.16	0.55 ± 0.17	0.74 ± 0.09	1.14 ± 0.26	1.02 ± 0.23
Opto-GPR176	0.53 ± 0.12	0.64 ± 0.12	0.96 ± 0.2	0.72 ± 0.13	0.67 ± 0.22	0.65 ± 0.14	0.91 ± 0.06	1.02 ± 0.12
Opto-GPR182	0.71 ± 0.18	0.75 ± 0.32	0.70 ± 0.11	1.72 ± 0.96	0.52 ± 0.15	0.56 ± 0.11	0.98 ± 0.07	1.13 ± 0.12
Opto-GPR183	0.43 ± 0.09	0.65 ± 0.07	0.88 ± 0.16	2.02 ± 1.01	0.76 ± 0.23	0.68 ± 0.18	0.96 ± 0.08	0.91 ± 0.11

Supplementary Table 8. Substitutions in fourteen Class A GPCRs. Receptors with at least one non-conservative substitution in the E/DRY motif and at least one non-conservative substitution in conserved contacts involved in active and inactive states (3x46, 6x37 and 7x53) are listed. Non-conservative substitutions are highlighted in red. Residues not found in non-orphan Class A GPCRs are highlighted with a star (*). Colors denote side chain chemistry (blue: aliphatic, orange: aromatic, red: basic, green: acidic, purple: polar, grey: other).

Receptor	E/DRY motif			Conserved contacts		
	3x49	3x50	3x51	3x46	6x37	7x53
GPR27	T*	R	Y	V	F	C*
GPR62	A	R	Y	L	P	Y
GPR75	H	R	L*	I	C	Y
GPR84	G	R	Y	I	C	Y
GPR85	T*	R	Y	I	F	C*
GPR141	T*	R	Y	I	N	F
GPR148	H	T	Y	I	H*	P*
GPR149	N	F*	Y	V	A	V*
GPR151	V	C	F	V	L	F*
GPR152	D	R	C	L	A	C*
GPR153	H	R	M	L	T	L
GPR162	H	R	M	L	S	I
GPR173	T*	R	Y	I	F	C*
Class A consensus	Acidic (E/D)	Basic (R)	Aromatic (Y)	Aliphatic	Aliphatic	Aromatic

Supplementary Table 9. Oligonucleotide PCR primers utilized in this study. Restriction sites are underlined where applicable.

**Number**	**Sequence**
1	GATCGCGGCCGCACCATGAGGACTCTGAACACCTCTGC
2	GATCGGTACCTCAGGTTGGGTGCTGACCG
3	GATCGCGGCCGCACCATGGATCCACTGAATCTGTCCTG
4	GATCGGTACCTCAGCAGTGGAGGATCTTCAGG
5	GATCGCGGCCGCACCATGAACACTTCAGCCCCAC
6	GATCAAGCTTTCAGCATTGGCGGGAGGG
7	GATCGCGGCCGCACCATGAATAACTCAACAAACTCCTCTAACAATA
8	GATCAAGCTTTTACCTTGTAGCGCCTATGTTC
9	CACTATAGGGAGACACAAGCTGGCTAGC
10	GCTAGCCAGCTTGTGTCTCCCTATAGTG
11	AATGATACCGCGGGACCCACGCTCA
12	TGAGCGTGGGTCCCGCGGTATCATT
13	GAGGATTGGGAAGAGAATAGCAGGCATGC
14	GCATGCCTGCTATTCTCTTCCCAATCCTC
15	GATCTGGACGAAGAACATCAGGGGCTCG
16	CGAGCCCCTGATGTTCTTCGTCCAGATC
17	GATATTGCTGAAGAACTTGGCGGCGAATGG
18	CCATTCGCCGCCAAGTTCTTCAGCAATATC
19	TATTGGGCGCTCTTGCGCTTCCTCGCTCA
20	TGAGCGAGGAAGCGCAAGAGCGCCCAATA
21	CAGATCGGCGCGCCTTAAGAATTCCACCACACTGGACTAGT
22	GTCTAGGGCGCGCCGGCAGGCGCCACTTGGCTG
23	CAAATTGGCGCGCCTGCGGTGAGCAAGGGCGAGGAGGATAAC
24	GTCTAGGGCGCGCCCTTGTACAGCTCGTCCATGCCGCC
25	GCCCAGAAGATCGAGTGGCACGAGGGCAGCAGCGGCGATAGCCTCATGAACGGGACCGA
26	CTCGAAGATATCGTTCAGGCCGCCGCTGCTGCCGGCGAATACCAGGCAGAAGATGT
27	CAGATCATCGATACAGAAACCAGCCAAGTGGC
28	CAGATCATCGATGCGGAACTGCTTGTTCATCATG
29	GATCGCTAGCATGGCTTCCAAGGTG
30	GATCTCTAGAATTACTGCTCGTTC

Supplementary References

1. Engler, C. & Marillonnet, S. Golden Gate cloning. *Methods Mol. Biol.* **1116**, 119-131 (2014).
2. Atwood, B. K., Lopez, J., Wager-Miller, J., Mackie, K. & Straiker, A. Expression of G protein-coupled receptors and related proteins in HEK293, AtT20, BV2, and N18 cell lines as revealed by microarray analysis. *BMC Genomics* **12**, 14 (2011).
3. Airan, R. D., Thompson, K. R., Fenno, L. E., Bernstein, H. & Deisseroth, K. Temporally precise in vivo control of intracellular signalling. *Nature* **458**, 1025-1029 (2009).
4. Schmidt, J. *et al.* Selective orthosteric free fatty acid receptor 2 (FFA2) agonists: identification of the structural and chemical requirements for selective activation of FFA2 versus FFA3. *J. Biol. Chem.* **286**, 10628-10640 (2011).
5. Cusack, N. J. & Hourani, S. M. 5'-N-ethylcarboxamidoadenosine: a potent inhibitor of human platelet aggregation. *Br. J. Pharmacol.* **72**, 443-447 (1981).
6. Werner, P., Hussy, N., Buell, G., Jones, K. A. & North, R. A. D2, D3, and D4 dopamine receptors couple to G protein-regulated potassium channels in Xenopus oocytes. *Mol. Pharmacol.* **49**, 656-661 (1996).
7. Arnt, J., Bogeso, K. P., Hyttel, J. & Meier, E. Relative dopamine D1 and D2 receptor affinity and efficacy determine whether dopamine agonists induce hyperactivity or oral stereotypy in rats. *Pharmacol. Toxicol.* **62**, 121-130 (1988).
8. Pan, Z. Z. & Williams, J. T. Muscarine hyperpolarizes a subpopulation of neurons by activating an M2 muscarinic receptor in rat nucleus raphe magnus in vitro. *J. Neurosci.* **14**, 1332-1338 (1994).
9. Benson, J. A. Electrophysiological pharmacology of the nicotinic and muscarinic cholinergic responses of isolated neuronal somata from locust thoracic ganglia. *J. Exp. Biol.* **170**, 203-233 (1992).
10. Lebois, E. P. *et al.* Discovery and characterization of novel subtype-selective allosteric agonists for the investigation of M(1) receptor function in the central nervous system. *ACS Chem. Neurosci.* **1**, 104-121 (2010).
