# Supplementary material for: Optical functionalization of human Class A orphan G-protein-coupled receptors
Source: Nat Commun. 2018 May 16;9:1950. doi: 10.1038/s41467-018-04342-1 (PMC5956105; doi:10.1038/s41467-018-04342-1)
Supplement: Supplementary file 3 — Description of Additional Supplementary Files [file 41467_2018_4342_MOESM3_ESM.docx]

**Description of Additional Supplementary Files**

File Name: Supplementary Data 1

Description: Protein sequences for reference GPCR chimeric receptors.

File Name: Supplementary Data 2

Description: Nucleotide sequences for proteins of Supplementary Data 1. The genes are

available through Addgene.org.

File Name: Supplementary Data 3

Description: Protein sequences for orphan and understudied chimeric receptors.

File Name: Supplementary Data 4

Description: Nucleotide sequences for proteins of Supplementary Data 3. The genes are

available through Addgene.org.
